# Fruit and Vegetable Intake and Stomach Cancer among Male Adults: A Case-Control Study in Northern Viet Nam

**DOI:** 10.31557/APJCP.2020.21.7.2109

**Published:** 2020-07

**Authors:** Le Hong Phuoc, Khanpaseuth Sengngam, Toshio Ogawa, Nlandu Roger Ngatu, Shunya Ikeda, Tran Hieu Hoc, Pham Van Phu, Dinh Thi Minh, Le Tran Ngoan

**Affiliations:** 1 *Graduate School of Public Health, International University of Health and Welfare, Narita city, Chiba Prefecture, Japan. *; 2 *Faculty of Public Health, University of Medicine and Pharmacy at Ho Chi Minh city, Ho Chi Minh city, Viet Nam. *; 3 *Hanoi Medical University, Ha Noi, Viet Nam. *; 4 *Department of Public Health, School of Medicine, International University of Health and Welfare, Narita city, Chiba prefecture, Japan. *; 5 *Institute of Research and Development, Duy Tan University, Da Nang city, Vietnam. *

**Keywords:** Stomach cancer, vegetable, fruit, Helicobacter pylori, tobacco smoking

## Abstract

**Objective:**

This study investigated the association between fruit and vegetable intake and stomach cancer, with considering the impacts of *Helicobacter pylori* (*H. pylori*) infection and tobacco smoking.

**Methods:**

A case-control study featuring 80 male incident stomach-cancer cases and 146 male controls was conducted in a general hospital in Viet Nam. A semi-quantitative food frequency and demographic lifestyle questionnaire were used; and venous blood samples were collected to determine *H. pylori *status by IgG ELISA. The respective associations between fruit and vegetable intake and stomach cancer were examined using unconditional logistic regression analysis with adjustments for possible cofactors.

**Results:**

Fruit intake and stomach cancer showed a weak inverse association when this became non-significant after adjusting for *H. pylori *infection (OR = 0.50, 95%CI: 0.22–1.12, p trend = 0.094). Stratifying by *H. pylori *status returned a negative trend for fruit intake and stomach cancer among *H. pylori*-negative participants (OR = 0.21, 95%CI: 0.06–0.69, p trend = 0.010), but no significant interaction for *H. pylori*-positive participants (OR = 0.76, 95%CI: 0.21–2.68, p trend = 0.670). Vegetable intake and stomach cancer showed no association, regardless of *H. pylori *status. Compared to ever-smokers with low intake, never-smokers with high vegetable (OR = 0.25, 95% CI: 0.06–0.95) and fruit intake (OR = 0.20, 95%CI: 0.06–0.65) showed the lowest odds of stomach cancer.

**Conclusions:**

Fruit, but not vegetable, intake showed a weak inverse association with stomach cancer. *H. pylori *infection and tobacco-smoking status may influence the protective effects of fruit and vegetable intake on stomach cancer.

## Introduction

Globally, stomach cancer is the sixth most-diagnosed cancer, and the third most common cause of cancer death (Bray et al., 2018). The contributions of dietary factors to stomach cancer have been documented in numerous studies; in particular, high intake of salt and salt-preserved foods (Wang et al., 2009), pickles, cured and smoked foods (Ren et al., 2012), and processed meats (González et al., 2006) have been found to increase the risk of stomach cancer, while fruits and vegetables have been found to have protective factors in this regard (Correa, 2013). However, to date there has been no clear finding regarding the benefits of fruits and vegetables for stomach cancer (Lunet et al., 2005; Tsugane and Sasazuki, 2007; World Cancer Research Fund/American Institute for Cancer Research, 2018). 

Chronic infection with *H. pylori *has also been found to be a carcinogen causing stomach cancer, and studies have suggested that fruit and vegetable intake modifies the effect of *H. pylori *infection on stomach cancer. Specifically, extracts from fruits and vegetables have been found to affect *H. pylori *infection in various methods, showing anti-*H. pylori*, anti-urease, anti-adhesive, and anti-invasive activities, as well as attenuating inflammation (Liu et al., 2018). Conversely, *H. pylori *infection may reduce the bioavailability of many anticarcinogenic vitamins and minerals widely found in fruits and vegetables (Zhang et al., 2002; Kim et al., 2005; Franceschi et al., 2014). The differing effects of fruits and vegetables on *H. pylori*-associated stomach cancer have been explored in some previous studies, but inconsistent results have been reported (Machida-Montani et al., 2004; Epplein et al., 2008; Wang et al., 2012; Wang et al., 2017). The above findings suggest that *H. pylori *infection plays a crucial role as a confounder in the relationship between fruit and vegetable intake and stomach cancer. As with *H. pylori *infection, tobacco smoking is carcinogenic to humans and several studies have suggested that tobacco smoking status impacts the effect of fruits and vegetables on stomach cancer (Nouraie et al., 2005; Epplein et al., 2010). 

The present study aimed to analyse the association between fruit and vegetable intake and stomach cancer among Vietnamese men, with adjustments for two well-known risk factors: *H. pylori *infection and tobacco smoking. 

## Materials and Methods

*Study design and participants*

**Figure 1:**
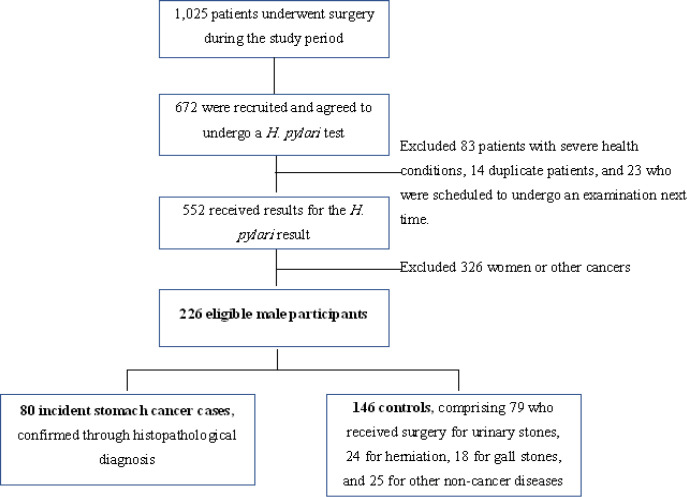
Participant Recruitment Process

A hospital-based case-control study was conducted between January and December 2018 in a general hospital located in northern Viet Nam. The cases comprised Vietnamese men who had been newly diagnosed with stomach cancer through histopathology, and who had undergone surgery for cancer treatment. Controls included male Vietnamese patients who had no history of cancer (at any site), and who underwent surgery in the same department as the cases during the same period. Exclusion criteria included 1) being on a diet, and 2) having a severe health condition or other morbidity that affected dietary patterns. Based on the list of patients who had description of surgery each week during the study period, 672 patients were chosen and agreed to undergo a *H. pylori *test. Of these participants, 326 patients who were women or who were identified as having other cancers. Additionally, 120 were excluded because they had severe health conditions (n = 83), were duplicate patients (n = 14; i.e., patients who were recruited on separate occasions by different interviewers), or were scheduled to undergo a *H. pylori*-examination the next time (n = 23). A total of 226 eligible male participants were recruited for this analysis, comprising 80 incident stomach-cancer cases and 146 controls (Figure 1).

*Data collection*

Participants were interviewed by trained interviewers on the day immediately before his surgery using a validated semi-quantitative food frequency and demographic lifestyle questionnaire. This questionnaire obtained information regarding demographic characteristics, family history of cancer (within second-degree relatives); dietary factors, and lifestyle factors (including tobacco smoking and alcohol consumption). For tobacco smoking, all participants were categorised into three groups: current, ex, and non-smokers, respectively. Current smokers were individuals who had completely consumed one tobacco product (through a cigarette or waterpipe tobacco) within the last six months; individuals who were not current smokers but had consumed tobacco products in their lifetime were classified as ex-smokers. Current and ex-smokers were then classified as ‘ever smokers’, and non-smokers as ‘never smokers’. In terms of alcohol consumption, participants were classified into the ‘ever drinkers’ category if they had consumed at least one serving of alcohol in one sitting within the last 12 months; all other individuals were allocated to the ‘never drinkers’ group.

Regarding dietary factors, all participants were asked, based on a validated semi-quantitative food frequency questionnaire (SQFFQ), about their average frequency of intake and the portion size over the last 12 months for 15 and 14 different types of vegetables and fruits, respectively. The SQFFQ used comprised 85 foods/recipes that cumulatively contribute up to 90% of the major nutrients; it was based on a database created from a 24-hour recall study conducted on 300 general households in Northern Viet Nam in 2017 (Le et al., 2018). In the questionnaire, respondents ranked the fruits and vegetables in terms of frequency of intake using a seven-point scale (from ‘seldom or never intake’ to ‘1–3 times/day’) and in terms of portion size using a three-point scale (‘small’, ‘medium’, and ‘large’). Daily intake for each fruit and vegetable (in grams) was estimated by multiplying the frequencies, portion sizes (small = 0.8, medium = 1, large = 1.2), and the average weights of the intake for the respective foods (minus the percentage of weight lost due to preliminary preparation, based on the Vietnamese food composition table, version 2007). Total fruit and vegetable intake (gram/day) were calculated by adding the amounts for all fruits and vegetables, respectively. Then, this total amount was divided into categories based on the nearest tertile; these tertiles were determined based on the distribution of intake among the controls.

To investigate the presence of *H. pylori *infection, 3-ml aliquots of venous blood (taken after overnight fasting) were obtained from each case and control. Anti-*H. pylori *serum IgG antibody titers were tested using an enzyme-linked immunosorbent assay (ELISA), based on the sandwich principle, through the use of a *H. pylori *IgG ELISA kit (RE56381; IBL International, Hamburg, Germany). *H. pylori *status was classified into three groups, based on a cut-off index (COI) provided by the manufacturer: negative (COI < 0.8), equivocal (0.8–1.2), and positive (> 1.2).

*Ethics*

The present study was approved by the ethics committees of Hanoi Medical University, Viet Nam and the International University of Health and Welfare, Japan. Additionally, written informed consent were obtained from all participants.

*Data analysis*

The collected data were analysed using Stata version 15.0 (Stata Corp, College Station, Texas). Unconditional logistic regression analysis, with adjustments for age, education level, family history of cancer, tobacco smoking, alcohol consumption, and *H. pylori *status was used to estimate the odds ratios (ORs) and 95% confidence intervals (95% CIs) for the association between fruit and vegetable intake and stomach cancer. To examine whether the relationship between fruit and vegetable intake and stomach cancer is affected by *H. pylori *status, all patients with equivocal results in the anti-*H. pylori *test were categorised into the negative group, creating two *H. pylori *serostatus groups: ‘negative’ and ‘positive’, respectively. Then, ORs and 95%CIs for the relationship between each exposure were calculated for these two strata of *H. pylori *status. All p-values were two-sided, and p ≤ 0.05 (alpha value) was considered to indicate statistical significance.

## Results

The mean ages of the cases and controls were 59.99 ± 11.72 and 54.54 ± 11.64 years, respectively (data not shown). Among the cases, 52.5% and 36.3% were current- and ex-smokers, respectively, while these figures among controls were 45.9% and 28.1%, respectively. Among the 226 participants, 38.9% (n = 88), 18.6% (n = 42), and 42.5% (n = 96) were classified as negative, equivocal, and positive, respectively, regarding *H. pylori *status. The cases showed a greater percentage of *H. pylori*-positive members when compared to the controls (50.0% versus 38.3%) (Table 1). The estimated amount of intake (median [interquartile range], grams/day) of the cases and controls were 85.72 [75.73–116.74] and 91.26 [72.58–122.65] for vegetables; and 67.12 [40.77–89.84] and 81.93 [59.65–101.62] for fruits, respectively (data not shown).

The results shown in Table 2 suggest an inverse association between fruit intake and stomach cancer. In Model 1, a significant negative trend between fruit intake and stomach cancer was indicated; this suggests that increased fruit intake is associated with lower odds of stomach cancer (p for trend = 0.046). Compared to the first tertile for fruit intake, the second and third tertiles showed lower odds of stomach cancer (OR = 0.72, 95%CI: 0.36–1.46; and OR = 0.45, 95%CI: 0.20–0.98, respectively). However, in Model 2, which additionally adjusted for *H. pylori *status, the negative relationship between fruit intake and stomach cancer was no longer statistically associated; this was despite the fact that a decreasing trend was still observed across the three tertiles of fruit intake (tertile 3 versus tertile 1: OR = 0.50, 95%CI: 0.22–1.12; p for trend = 0.094). No association between vegetable intake and stomach cancer was found in either model.

After stratifying by *H. pylori *status (Table 3), a significant negative association between fruit intake and stomach cancer was observed among *H. pylori*-negative individuals (p for trend = 0.010), but no such statistically significant relationship was found among *H. pylori*-positive individuals (p for trend = 0.670). For *H. pylori*-negative participants, the odds of stomach cancer decreased across the fruit intake tertiles, with the ORs among the second and third tertiles being 0.49 (95%CI: 0.17–1.36), and 0.21 (95%CI: 0.06–0.69), respectively. No statistically significant association between vegetable intake and stomach cancer was observed, regardless of *H. pylori *status.

Regarding the joint effect of tobacco smoking and fruit and vegetable intake, compared to ‘ever smokers’ (i.e., current- and ex-smokers) and who had low vegetable intake, ‘never smokers’ who had a high vegetable intake showed the strongest inverse association between vegetable intake and stomach cancer (OR = 0.25, 95%CI: 0.06–0.95; meanwhile, no statistically significant difference was observed for ‘ever smokers’ and high vegetable intake (OR = 1.23, 95%CI: 0.63–2.40; Table 4). Similarly, compared to ever smokers with low fruit intake, never smokers with high fruit intake also showed the lowest odds of stomach cancer (OR = 0.20, 95%CI: 0.06–0.65).

**Table 1:** Participants’ Characteristics

	Cases (n = 80)	Controls (n = 146)	p^a^
	N (%)	N (%)	
Age group (years)			0.058
20–29	1 (1.3)	4 (2.7)	
30–39	3 (3.7)	12 (8.2)	
40–49	10 (12.5)	31 (21.2)	
50–59	24 (30.0)	53 (36.3)	
60–69	28 (35.0)	31 (21.2)	
≥ 70	14 (17.5)	15 (10.3)	
Education level			0.231
Primary school	16 (20.0)	19 (13.0)	
Secondary school	36 (45.0)	64 (43.8)	
High school or higher	28 (35.0)	63 (43.2)	
Family history of cancer			0.238
Yes	7 (8.8)	7 (4.8)	
No	73 (91.2)	139 (95.2)	
Tobacco smoking			0.009
Current	42 (52.5)	67 (45.9)	
Ex	29 (36.3)	41 (28.1)	
Never	9 (11.2)	38 (26.0)	
Alcohol consumption			0.034
Ever	71 (88.8)	108 (74.0)	
Never	9 (11.2)	38 (26.0)	
*H. pylori* status			0.062
Negative	25 (31.3)	63 (43.2)	
Equivocal	15 (18.7)	27 (18.5)	
Positive	40 (50.0)	56 (38.3)	

^a^ *p*-value generated through a chi-square test

**Table 2.** Adjusted Odds Ratios and 95% Confidence Intervals for the Relationship between Vegetable Consumption, Fruit Consumption and Stomach Cancer

	Cases (n = 80) N (%)	Controls (n = 146) N (%)	Model 1	Model 2
			OR (95%CI)^a^	OR (95%CI)^b^
Vegetable intake (grams/day)				
1 (< 78.55)	28 (35.0)	48 (32.88)	1.00 (reference)	1.00 (reference)
2 (78.55–108.83)	28 (35.0)	47 (32.19)	1.40 (0.67–2.93)	1.28 (0.60–2.73)
3 (> 108.83)	24 (30.0)	51 (34.93)	1.20 (0.56–2.56)	1.14 (0.53–2.46)
p for trend			0.637	0.736
Fruit intake (grams/day)				
1 (< 62.97)	33 (41.3)	43 (29.4)	1.00 (reference)	1.00 (reference)
2 (62.97–89.99)	28 (35.0)	47 (32.2)	0.72 (0.36–1.46)	0.77 (0.37–1.57)
3 (> 89.99)	19 (23.7)	56 (38.4)	0.45 (0.20–0.98)	0.50 (0.22–1.12)
p for trend			0.046	0.094

^a^Estimates were adjusted for age (years, continuous), education levels (primary school, secondary school, high school or higher, unknown), family history of cancer (yes, no), tobacco smoking (current, ex, never), alcohol consumption (ever, never), and fruit or vegetable intake (whenever one was not the main exposure); ^b^Estimates were adjusted for age (years, continuous), education levels (primary school, secondary school, high school or higher, unknown), family history of cancer (yes, no), tobacco smoking (current, ex, never), alcohol consumption (ever, never), fruit or vegetable intake (whenever one was not the main exposure), and *H. pylori *serostatus (negative, equivocal, positive).

**Table 3:** Adjusted Odds Ratios and 95% Confidence Intervals for the Relationship between Vegetable Consumption, Fruit Consumption, and Stomach Cancer in Terms of *H. pylori* Infection Status

	*H. pylori* infection (+)			*H. pylori* infection (-)		
	Cases	Controls	Adjusted^a^	Cases	Controls	Adjusted^a^
	(n = 40) N (%)	(n = 56) N (%)	OR (95% CI)	(n = 40) N (%)	(n = 90) N (%)	OR (95% CI)
Vegetable intake						
Tertile 1	12 (30.0)	20 (35.8)	1.00 (reference)	16 (40.0)	28 (31.1)	1.00 (reference)
Tertile 2	14 (35.0)	18 (32.1)	1.25 (0.35–4.46)	14 (35.0)	29 (32.2)	1.37 (0.48–3.86)
Tertile 3	14 (35.0)	18 (32.1)	1.83 (0.51–6.59)	10 (25.0)	33 (36.7)	0.97 (0.31–2.98)
p for trend			0.348			0.974
Fruit intake						
Tertile 1	15 (37.5)	17 (30.4)	1.00 (reference)	19 (47.5)	25 (27.8)	1.00 (reference)
Tertile 2	13 (32.5)	19 (33.9)	0.61 (0.18–2.05)	14 (35.0)	29 (32.2)	0.49 (0.17–1.36)
Tertile 3	12 (30.0)	20 (35.7)	0.76 (0.21–2.68)	7 (17.5)	36 (40.0)	0.21 (0.06–0.69)
p for trend			0.670			0.010

^a^Estimates were adjusted for age (years, continuous), education levels (primary school, secondary school, high school or higher, unknown), family history of cancer (yes, no), tobacco smoking (current, ex, never), fruit or vegetable intake (whenever one was not the main exposure).

**Table 4:** Multivariate Odds Ratios and 95% Confidence Intervals for the Joint Effects of Fruit and Vegetable Intake and Tobacco Smoking on Stomach Cancer

		Cases (n = 80) N	Controls (n = 146) N	Unadjusted	Adjusted^b^
				OR (95% CI)	OR (95% CI)
Tobacco smoking	Vegetable intake^a^				
Ever	≤ 50% percentile	37	56	1.00 (reference)	1.00 (reference)
	> 50% percentile	34	52	0.99 (0.54–1.80)	1.23 (0.63–2.40)
Never	≤ 50% percentile	6	14	0.65 (0.23–1.84)	0.63 (0.21–1.87)
	> 50% percentile	3	24	0.19 (0.05–0.67)	0.25 (0.06–0.95)
Tobacco smoking	Fruit intake^a^				
Ever	≤ 50% percentile	42	56	1.00 (reference)	1.00 (reference)
	> 50% percentile	29	52	0.74 (0.41–1.36)	0.81 (0.42–1.57)
Never	≤ 50% percentile	5	10	0.67 (0.21–2.10)	0.63 (0.19–2.15)
	> 50% percentile	4	28	0.19 (0.06–0.58)	0.20 (0.06–0.65)

^a^Fruit and vegetable intake were categorised into two: low intake (< 50% percentile) and high intake (≥ 50% percentile) based on the distribution of intake among controls; ^b^Estimates were adjusted for age (years, continuous), education levels (primary school, secondary school, high school or higher, unknown), family history of cancer (yes, no), alcohol consumption (ever, never), fruit or vegetable intake (whenever one was not the main exposure), and *H. pylori* serostatus (negative, equivocal, positive)

## Discussion

The present study findings suggest that there is a weak inverse association between fruit intake and stomach cancer, and that *H. pylori *infection potentially has a modifying effect on this association. After additional adjustment for *H. pylori *status, the negative relationship between fruit intake and stomach cancer became non-significant (Model 2). Moreover, sub-analysis stratifying by *H. pylori *status (positive and negative) returned a significant association between fruit intake and stomach cancer among *H. pylori*-negative patients, but not among *H. pylori*-positive patients. Prior studies have also suggested that there is a weak inverse association between fruit intake and stomach cancer; several case-control studies (Lee et al., 2003; Lunet et al., 2007; Wang et al., 2012; Wang et al., 2017) have reported a significant negative association, while most cohort studies have not found a significant association (Larsson et al., 2006; Freedman et al., 2008; Steevens et al., 2011; Shimazu et al., 2014; World Cancer Research Fund/American Institute for Cancer Research, 2018). Regarding the joint effect of fruit intake and *H. pylori *infection on stomach cancer, the results of the present study are somewhat consistent with at least three comparable previous studies: one conducted in China (Wang et al., 2012), one in Japan (Machida-Montani et al., 2004), and a joint analysis conducted in China, Japan, and Korea (Wang et al., 2017). The first is a case-control study among a Chinese population (Wang et al., 2012) that reported a significant interaction between *H. pylori *infection and fruit intake in regard to non-gastric cancer (p for interaction = 0.011). Although two later studies (the above-mentioned Japan-based study and the joint analysis) found no significant interaction in this regard they, nevertheless, indicated that *H. pylori*-positive individuals with low fruit intake have the greatest odds of stomach cancer (Machida-Montani et al., 2004; Wang et al., 2017). Reduced bioavailability and malabsorption of essential nutrients in fruits and vegetables may be a consequence of *H. pylori *infection; for example, *H-pylori*-positive individuals show decreased concentration of β-carotene in gastric juice, even when they have similar plasma levels as uninfected controls (Zhang et al., 2000). Moreover, the bioavailability of other substances that can protect against stomach cancer, such as vitamin A, vitamin C, vitamin E, and β-carotene, may also be reduced by *H. pylori *infection (Zhang et al., 2002; Kim et al., 2005; Franceschi et al., 2014). The above evidence suggests that *H. pylori *infection modulates the association between fruit intake and stomach cancer, weakening the protective effects of fruits for individuals with *H. pylori *infections. 

The present findings indicate a null association between vegetable intake and stomach cancer, regardless of *H. pylori *status. There is, as yet, no consensus regarding the relationship between vegetable intake and stomach cancer. Two recent large cohorts (Gonzalez et al., 2012; Shimazu et al., 2014) have reported a marginally or non-significant association. Similarly, an updated report by the World Cancer Research Fund/American Institute for Cancer Research (World Cancer Research Fund/American Institute for Cancer Research, 2018) concluded that there is limited evidence (and no conclusion) regarding the association between the intake of vegetables and stomach cancer. This relationship, particularly in Asia and developing countries, should be investigated with caution, as a result of the high prevalence of *H. pylori *infections and the cooking methods used. The vegetable-consumption and cooking habits (i.e., boiling, frying, steaming) of Asian countries, including Viet Nam, may reduce the benefits of vegetable intake. In fact, a previous study suggested that there is a stronger inverse association between raw vegetables and cancers, particularly cancers of the upper gastrointestinal tract, when compared to cooked vegetables (Link and Potter, 2004). However, it should also be noted that epidemiological studies have suggested that foods, including fresh vegetables, play a role in the transmission of *H. pylori *(Quaglia and Dambrosio, 2018). 

Regarding the influence of tobacco-smoking status on the effects of fruits and vegetables on stomach cancer, the present study observed that high intake of fruits and/or vegetables is related to significantly lower odds of stomach cancer among never-smokers, but not among ever-smokers (compared to low-intake ever-smokers). Several studies have indicated that concentrations of β-carotene and vitamin C are significantly lower among smokers than non-smokers (Jarosz et al., 2000; Lykkesfeldt et al., 2000; Galan et al., 2005; Schleicher et al., 2009; Sugiura et al., 2009); moreover, in one study smokers showed lower plasma concentrations of these micronutrients than non-smokers, even though both groups had consumed the same amount (Northrop-Clewes and Thurnham, 2007). Therefore, the present findings support the hypothesis that tobacco smoking is associated with a reduction in the stomach-cancer-associated benefit obtained from fruits and vegetables.

The present study has several limitations. First, the case-control design of this study is susceptible to reverse causation and recall bias. Information on food intake was collected for the period of 12 months preceding the interview; this relatively long duration may have made it difficult for participants to accurately report their fruit and vegetable intake habits. However, the SQFFQ was used in the present study, which has shown reasonable validity and reliability regarding capturing dietary information among the Vietnamese population when compared to 24-hour diet recall records (Le et al., 2018). Additionally, it is possible that the cases, as a result of their cancer diagnosis, changed their dietary habits to more frequent intake of fruits and vegetables; this may have led to an underestimation of the association between fruit and vegetables and stomach cancer. Therefore, a caution on the interpretation of the causal relationship between exposures and stomach cancer should be considered. Second, the recruitment of hospital-based controls may have contributed to bias in dietary assessment and might not reflect the dietary habits of the general population. Third, the small sample size used also is an important drawback that weakens the examined associations between the target exposures and stomach cancer. This may be the potential for observed non-significant associations including resulting in the false-negative for the relationship between fruit intake and stomach cancer (Model 2).

In conclusion, the present study suggested that there is a weak inverse association between fruit intake and stomach cancer, but no such association for vegetables. In addition, *H. pylori *infection and tobacco smoking appear to be important confounders for the relationship between fruit and vegetable intake and stomach cancer, attenuating the associated benefits provided by these foods.
